# Genetic Diversity of *Venturia inaequalis* in Latvia Revealed by Microsatellite Markers

**DOI:** 10.3390/pathogens11101165

**Published:** 2022-10-09

**Authors:** Olga Sokolova, Inga Moročko-Bičevska, Gunārs Lācis

**Affiliations:** 1 Institute of Horticulture, Latvia University of Life Sciences and Technologies, LV-3701 Dobele, Latvia; 2 Institute of Soil and Plant Sciences, Latvia University of Life Sciences and Technologies, Lielā str. 2, LV-3001 Jelgava, Latvia

**Keywords:** apple scab, fungi, plant pathogens, population, SSR

## Abstract

Apple scab caused by the ascomycete *Venturia inaequalis* is an economically significant disease worldwide. The annual sexual reproduction of *V. inaequalis* leads to high variation, changes in the population’s genetic structure and adaptations to the changing environment, including overcoming the host’s resistance. The objective of this study is to characterise and assess the genetic diversity of *V. inaequalis* populations in two main apple-growing regions in Latvia. In total, 143 *V. inaequalis* isolates were collected from Latvia, six reference strains with known virulence were obtained from other countries, and all strains were genotyped by 12 SSR markers. The SSR markers were highly variable and informative, identifying 158 alleles that ranged from two to 29 per locus. The Bayesian clustering identified three genetic lineages among the Latvian isolates that did not correlate to the geographic origin, host genotype, organ (leaves or fruits) from which the pathogen was isolated, time of collection, and type of isolation (single conidium or ascospore). The possible relatedness to virulence was detected when reference strains with known virulence were included in the analysis. Our findings correspond with previous studies demonstrating that *V. inaequalis* in Europe has a high genetic diversity within populations, but low diversity among the populations.

## 1. Introduction

Apple scab caused by the ascomycete *Venturia inaequalis* (Cooke) is an economically significant disease that affects apples (*Malus × domestica* Borkh.) worldwide, causing deformations in fruit shape and size that may result in yield reductions of up to 70% [1]. In areas with favourable conditions for apple scab, mainly in countries with a temperate climate, with cool, moist weather in early spring, the disease can affect the quantity and quality of the yield, and up to 80–100% of susceptible varieties [2]. Fungicides are applied several times, even up to 15–20 times in conducive conditions, throughout the growing season in order to prevent the spread of the apple scab [3,4]. Although in orchards with resistant varieties, where the number of fungicide treatments is reduced [5], it can still build to a significant amount, thus leading to the build-up of resistance in the pathogen’s population [6]. In Latvia, traditionally, minimal amounts of fungicides have been used to control apple scab, usually targeting the primary infection period and ranging between two to eight sprays per season [7,8].

*V. inaequalis* is a heterothallic pathogen with annual sexual reproduction and multiple asexual reproductions. The annual sexual reproduction leads to high variation, changes in the genetic structure of the *V. inaequalis* population, and adaptations to the changing environment, including forming new races and overcoming host resistance [9]. Currently, 17 gene-for-gene relationships have been described for the *Malus* and *V. inaequalis* pathosystem, and 14 races of *V. inaequalis* virulent on host genotypes with corresponding resistant genes (*Rvi*) are known to occur in nature [10,11]. The characterisation of the *V. inaequalis* population is crucial for understanding the epidemiology of the apple scab, and it can provide growers with valuable information concerning disease management [9]. Studies on the genetic diversity of *V. inaequalis* have been conducted in several countries in central and western Europe [12,13,14], Israel [15], central Asia [16], Turkey [17], Iran [9], South Africa [18], United States [19], Belarus [20], and Russia [2]. Various genotyping techniques have been employed to analyse the genetic diversity of *V. inaequalis* populations, such as random amplified polymorphic DNA (RAPD) markers, restriction fragment length polymorphism (RFLP), genome-wide SNPs, as well as microsatellite or simple sequence repeat (SSR) markers [9,13,16,21,22,23,24]. SSRs markers are widely used to study genetic diversity in pathogen populations, as they are codominant, multiallelic, and provide a good level of population-level resolutions [25].

The information on the genetic structure of *V. inaequalis* in Latvia is lacking. Weather conditions in Latvia, where rain is prevalent from spring to late autumn, are conducive for apple scab development throughout the season. In addition, weather conditions during the dormancy period are usually suitable for forming the sexual stage of the pathogen, as it forms pseudothecia in leaf litter. In Latvia, a wide range of apple cultivars that are adapted to local climatic conditions are grown, including locally bred or introduced cultivars from several growing regions, such as Russia, Belarus, Lithuania, and Estonia. In the breeding of new cultivars, the donors of important agronomic traits and disease resistance from other countries and regions, such as Germany, Ukraine, Czechia, France, the United States, Canada, New Zealand, and Scandinavia, are used [26]. Although diverse varieties, instead of mono-cultivar orchards, may be beneficial for reducing the risk of formation of new races, disease epidemics, and fungicide treatments [27], they may also have adverse effects (e.g., introduction of new races (genotypes) of the pathogen from other growing regions with new host genotypes) [28]. The objective of this study is to characterise and assess the genetic diversity of *V. inaequalis* populations in Latvia in two main apple-growing regions using SSR markers.

## 2. Materials and Methods

### 2.1. Sampling and Fungal Isolation

Samples were collected from two main apple-growing regions in the central (Zemgale) and North-western (Kurzeme) parts of Latvia from 2010 to 2016, and they were treated as separate populations in the data analysis (Table 1). Apple leaves and fruits with typical scab symptoms were collected from trees of various ages in commercial orchards, germplasm collections, experimental orchards, greeneries, and home gardens. Sampling was conducted from 19 *Malus* genotypes, including locally bred Latvian cultivars and hybrids (e.g., Nr 29-97-1; Nr 16-97-109; ‘Stars’, ‘Rāja’, ‘Carnikava’, ‘Popes ābele’, ‘Rudens Svītrainais’), widely grown commercial cultivars (e.g., ‘Lobo’, ‘Belorusskoje Malinovoje’, ‘Auksis’, ‘Kovalenkovskoje’, ‘Rubin’ (Kazakhstan cv.)), other introduced cultivars (e.g., ‘Vista Bella’, ‘Sister of Liberty’), and the ornamental apple, *M. toringo*.

Single spore isolates were obtained by streaking spore suspensions on 3% water agar (WA; Difco) containing streptomycin (50 μg/mL) to avoid bacterial contaminations. Single germinated spores were excised, transferred on potato dextrose agar (PDA, Difco), and isolates were preserved in 7 mL tubes at +5 ± 1 °C on PDA (Difco) and potato–carrot agar slants (PCA; [29]), and in the sterile distilled water for further cultivation. In total, 143 *V. inaequalis* strains originating from diverse localities and hosts in Latvia were genotyped. In addition, six *V. inaequalis* reference strains 1127, 147, 333, 1634, EU-B04, and 2408 for the races (1), (2), (4), (5), (6), and (8), which were found to be virulent on hosts with the resistance genes *Rvi1*, *Rvi2*, *Rvi4*, *Rvi5*, *Rvi6,* and *Rvi8* ([10,30], V. Caffier, personal communication for strain 333), respectively, were also included.

### 2.2. DNA Extraction

Fungal isolates were grown in Petri dishes containing 25 mL of potato dextrose broth (PDB; Difco) for 21 to 28 days at room temperature (22 ± 2 °C) without shaking. The mycelia were harvested without agar plugs, placed in 50 mL Falcon tubes, washed three times with sterile distilled water, and harvested via centrifugation at 3.500× rpm for 5 min (5430R, Eppendorf). The harvested mycelium was freeze-dried (FreeZone, Labconco) and ground to a fine powder in liquid nitrogen using a mortar and pestle. DNA was extracted using a DNeasy Plant Mini kit (Qiagen), in accordance with the manufacturer’s instructions. The concentration of each DNA sample was determined on a spectrophotometer (NanoDrop^®^ ND-1000, Nanodrop Technologies).

### 2.3. PCR Amplification

Initially, 31 published SSR primer pairs [21,31,32] were tested on selected eight strains from Latvia and five reference strains, representing diverse localities and host cultivars (data not shown). Twelve markers were selected for further analysis as they were ascertained to be the most informative and reliable in terms of variable allele size, single amplicon, and repeatability in replicate PCRs (Table 2, Figure S1).

The forward primers labelled with fluorescent dyes were used. The PCR amplifications were performed as singleplex assays for each marker at a 25 µL reaction volume with Type-it Microsatellite PCR Kit (Qiagen), in accordance with the manufacturer’s instruction. The PCR amplifications were performed with an Eppendorf Mastercycler EP Gradient thermocycler under the following conditions: 5 min at 95 °C, followed by 35 cycles of 30 s at 95 °C, 90 s at 58 °C (or 60 °C depending on the marker), and 30 s at 72 °C, with a final extension of 30 min at 60 °C. The annealing temperature used for each SSR primer pair is shown in Table 2.

The amplification products during the primer tests were separated by electrophoresis in 4% agarose gel (Applichem), stained with ethidium bromide, and visualised under UV light (AlphaDigiDocTMRT, Syngene). The approximate sizes of the amplified fragments were estimated with an O’RangeRuler 20 bp DNA Ladder (Thermo Fisher Scientific). The final sizing of PCR fragments was performed via capillary electrophoresis with a 3130xl Genetic Analyzer ABI (Applied Biosystems) as an external service at Latvia State Forest Research Institute “Silava”.

### 2.4. Data Analysis

The software GenAlEx 6.41 [33] was used to estimate genetic diversity parameters for each microsatellite locus: the number of alleles per locus, private alleles, allele frequencies, the effective number of alleles, Shannon’s index of diversity, and gene diversity. The polymorphic information content (PIC) was calculated using the computer programme SSRs written by W.F. Lamboy [34]. AMOVA analysis, including the calculation of the coefficient of genetic differentiation among populations (PhiPT), was also performed by GenAlEx with 999 permutations to examine the distribution of the variation and differential connectivity among populations. The principal component analysis (PCA) and discriminant analysis of principal components (DAPC) were carried out using PAST 3v1.0 software [35] to assess the variation of *V. inaequalis* multilocus genotypes. Before PCA analysis, the genotyping data were transformed in a binary format into a data matrix file, scoring the presence of an allele in each locus as “1” and its absence as “0”.

For population structure analysis, Bayesian model-based clustering program STRUCTURE version 2.3.4. [36] was used to detect the most likely number of populations (K) among the *V. inaequalis* isolates based on allele frequencies per locus. Each run consisted of a burn-in period of 100,000 steps, followed by 100,000 Monte Carlo Markov Chain replicates, assuming an admixture model and correlated allele frequencies. No prior information was used for a cluster definition. The ad hoc measure of ΔK was applied to calculate the optimum number of populations (K) [37] using the online software, Structure Harvester version 0.6.94 [38]. Twenty simulation runs with the highest modal value of ΔK were aligned in the Clumpp 1.1 cluster matching and permutation software [39], and they were visualised using Structure Plot V2.0 [40].

## 3. Results

Initially, 31 previously published SSR markers [21,31,32] were examined on the test group of *V. inaequalis* strains. Nineteen markers were excluded from the further analysis because 11 markers (1aac3b; 1aac4h; Vigt8/146; Viaggt8/1; Vitcca7/P; Vitc2/16; Vitg9/99; Vica9/x; ViaacS10; Vica9/134; Vitg9/129) were monomorphic, six (1aac4b; 1aac4f; EMVi001b; EMVi0032c; Viga3/z; Vica10/154) gave several amplification bands, and two markers (Vitc1/82, Vitc1/2) did not have any amplification product in ten of the tested strains (Data not showed).

In total, 143 *V. inaequalis* isolates were genotyped in 12 SSR loci. All these markers were polymorphic and multiple alleles were noted (Table 3). One hundred fifty-eight alleles, ranging from two to twenty-nine per locus, were found (Table 3). Markers Vigt 10/ε and Vict 1/130 noted the least allelic polymorphism, identifying two and five alleles per locus, respectively. The greatest polymorphism was revealed by the Vitc2/D (29 alleles per locus) and 1tc1g (27 alleles per locus) markers. In the other loci, the number of alleles varied considerably, depending on the marker (Table 3). The effective number of alleles (N_e_) at each locus ranged from 1.01 to 14.31. Nei’s gene diversity index (h) ranged from 0.01 to 0.93. Shannon’s information index (I) ranged from 0.04 to 2.88, and the number of private alleles ranged from one (Vigt 10/ε and Vict 1/130) to 15 (1tc1g). Polymorphic information content (PIC) values ranged from 0.013 (Vigt10/ε) to 0.934 (Vitc2D). Vict1/130 marker (PIC = 0.405) had moderate polymorphism (0.25 < PIC < 0.5), whereas the other ten loci (PIC = 0.549–0.934) had high polymorphism (PIC > 0.5) (Table 3).

**Figure 1:**
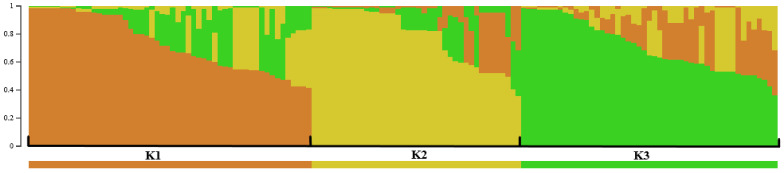
Population structure of *V. inaequalis* in two apple growing areas in Latvia, uncovered by the program STRUCTURE, based on the genotyping data of 143 isolates within twelve microsatellite loci and without prior information for the cluster definition. Each colour represents the genetic clusters identified (K = 3). Each individual isolate is represented by a vertical line in a bar plot. The individuals with multiple colours have admixture genotypes from multiple clusters.

**Figure 2:**
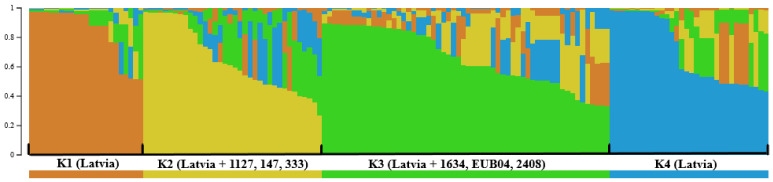
Population structure of *V. inaequalis* in two apple growing areas in Latvia, uncovered by the program STRUCTURE, based on the genotyping data of 143 isolates from Latvia and six reference strains with known virulence from other countries within twelve microsatellite loci and without prior information for the cluster definition. Each colour represents the genetic clusters identified (K = 4). Each individual isolate is represented by a vertical line in a bar plot. The individuals with multiple colours have admixture genotypes from multiple clusters.

The PCA analysis did not reveal any apparent clustering of the strains related to the initially defined populations based on the growing region (Zemgale and Kurzeme), specific orchard, or host genotype (data not shown). Therefore, for *V. inaequalis* population structure analysis, the program STRUCTURE was used to reveal populations (K) based on the allele frequencies per locus without prior information for the cluster definition. The population structure analysis, including isolates that were only from Latvia, identified three divergent groups, K1–K3 (Figure 1), whereas four groups (K1–K4) were recognised when six *V. inaequalis* reference strains with known races and characterised virulence were included (Figure 2).

The three genetic clusters revealed in the population structure analysis, including *V. inaequalis* isolates that were of Latvian origin, were not related to the initially defined populations based on the apple-growing regions (Zemgale and Kurzeme). The first group (K1) included 38% (n = 54) of genotyped *V. inaequalis* isolates that were derived equally from both regions (Zemgale and Kurzeme), and from the majority of the host genotypes (n = 11 + three unknown) represented in this study. In this group, the majority of the strains were monoconidial isolates (n = 46) that were obtained before 2013 (n = 44), in the two main fruit growing centres in Latvia (Dobele and Pūre; n = 44), from fruits (n = 33) in commercial orchards (n = 32) that had been subjected to fungicide treatments (n = 36). The second group (K2) comprised 28% (n = 40) of the strains, and they were mainly from Zemgale (82%; n = 33). The strains were collected on fruits or leaves in different years and from different types of orchards that had either been subjected to a fungicide treatment (n = 22) or had not (n = 18). Half of the strains in this group originated from commercial orchards (n = 20) and the cultivar ‘Lobo’ (n = 17). The third group (K3) included 34% (n = 49) of the *V. inaequalis* strains, of which 73% originated in Zemgale (n = 36; particularly, Dobele n = 31). The strains were obtained equally from fruits (n = 24) or leaves (n = 25), mainly as monoconidial isolates (n = 30) before 2013 (n = 33), and from various types of orchards that had (n = 21) or had not (n = 28) been subjected to fungicide treatment. Isolates in this group originated from 14 host genotypes, and half of them were from the cultivars ‘Huvitus’ (n = 10), ‘Beloruskoje Malinovoje’ (n = 9), and ‘Lobo’ (n = 5). The gene diversity (h) was comparable among the three genetic clusters (K), ranging from 0.53 to 0.64. Shannon’s information index (I), an estimate of diversity, ranged from 1.18 (K2) to 1.51 (K3) (Table 4).

The AMOVA results pointed to higher levels of variation within populations (87%) than between populations (13%). The low genetic differentiation among the populations in Zemgale and Kurzeme was also supported by a low PhiPT value. Higher PhiPT values (0.128–0.147) were detected among groups that were identified by STRUCTURE analysis (Table 5).

The markers Vitc2/D, 1tc1g, and 1tc1a revealed the greatest polymorphism in all groups, whereas markers Vicacg 8/42, Vitg 11/70, Vict 1/130, and Vigt 10/ε showed higher polymorphism in K3 than in other groups (Table 6). The identified effective number of alleles (N_e_) by the marker Vitc2/D was higher in K3 (12.84) than in K1 (8.84) and K2 (8.51), ranging from 1.04 to 12.84, depending on the locus and group. In K3, the gene diversity index (h) ranged from 0.04 to 0.92 depending on the marker, and it was higher than in other groups. In addition, Shannon’s information index (I) (0.10 to 2.72) and the number of private alleles (P_a_) (2.75) were also higher in K3 than in the other two groups (Table 4 and Table 6).

To understand the possible reasons for the grouping of the isolates, regardless of the growing region in Latvia (Kurzeme or Zemgale), specific orchard, or host genotype, six *V. inaequalis* reference strains with known races and characterised virulence [10,30] were included in the population structure analysis, in addition to the isolates from Latvia. The population structure analysis identified four divergent groups (Figure 2). The grouping was generally concordant with the first analysis (K1 = K2, K2 = K3, K4 = K1), except that the largest group, K3 (n = 58), was formed and equally included strains from all of the three groups (K1 = 17, K2 = 20, K3 = 18) from the first analysis, in addition to three reference strains, EU-B04, 1634, and 2408, which are known to be virulent on the *Rvi1*, *Rvi4*, and *Rvi8* hosts, respectively [10,30]. This group included strains from Kurzeme (n = 18) and Zemgale (37), and were mostly obtained as monoconidial isolates (n = 33) from fruits (n = 33) collected before 2013 (n = 47) in commercial orchards (n = 32), germplasm collection and related ornamental plantings (n = 17). The other three reference strains, 147, 1127, and 333, which are known to be virulent on hosts with the resistant genes *Rvi5*, *Rvi6* [10,30], and *Rvi2* (V. Caffier, personal communication), respectively, were included in K2 (n = 36). This group mainly comprised strains obtained as monoconidial (n = 14) or ascospore (n = 16) isolates from scabbed leaves that were collected in closely located and related (plant material exchange) orchards in Zemgale (n = 30), particularly Dobele (n = 28), and were mainly from areas with the fungicide-free growing system (n = 28). These two groups, K2 and K3, included the highest number of isolates from the greatest variety of genotypes (14 out of 19). Strains isolated before 2013 (K3 n = 47 out of 58; K4 n = 27 out of 32), as monoconidial isolates from fruits collected in the fungicide-treated orchards, were dominant in groups K3 and K4. The highest effective number of alleles (N_e_), the gene diversity index (h), and Shannon’s information index (I) were observed in groups K2 and K3, which included reference strains (Table 7 and Table 8). The number of private alleles (P_a_) was highest in K2 (2.00) and K3 (1.83) (Table 7).

AMOVA results showed that 85% of the genetic variation was distributed among individuals within populations, and only 15% of the variation was attributable to differences among the populations (Table 5). The greatest polymorphism was revealed by the marker Vitc2/D in groups K1, K3, and K4 (9, 19, and 10 alleles per locus, respectively) and 1tc1g (8/17/15/7 alleles per locus in groups K1, K2, K3, and K4) (Table 8). Vicacg8/42 showed high polymorphism (10 alleles per locus) in K2, but not in other groups.

**Figure 3:**
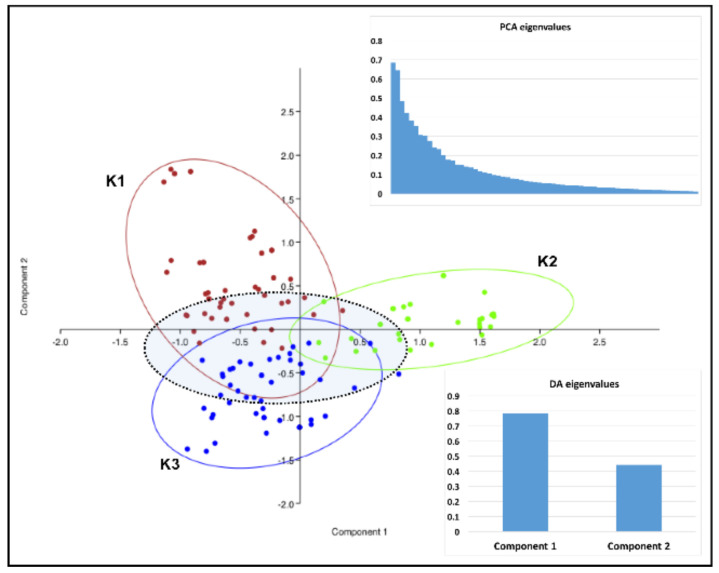
Scatter plot of the discriminant analysis of the principal components (DAPC) presenting interrelationships based on the genotyping data within twelve microsatellite loci of 143 *V. inaequalis* isolates sampled in two apple growing regions in Latvia. Coloured dots enclosed with 95% confidence ellipses represent isolates included in the genetic clusters (K = 3) that were identified in STRUCTURE analysis. The filled ellipse with a black dotted outline includes isolates that were sampled from each genetic cluster and placed in the cluster K3 during STRUCTURE analysis when the reference strains with known virulence were included. The graphs show the eigenvalues retained in discriminant analysis (DA) and principal component analysis (PCA).

**Figure 4:**
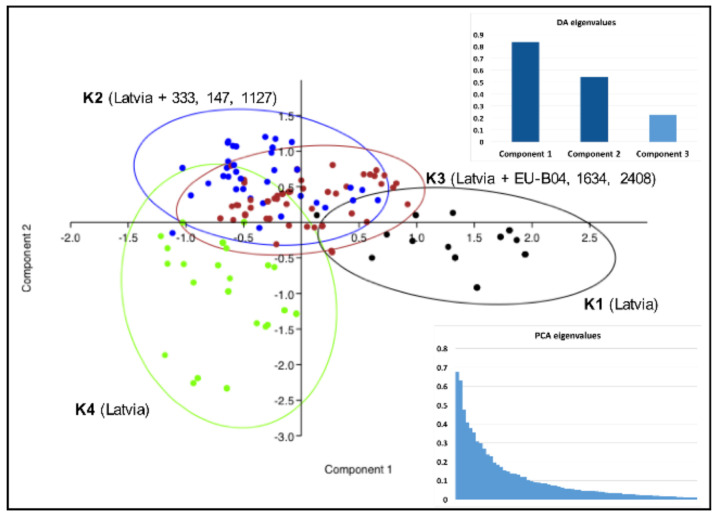
Scatter plot of the discriminant analysis of the principal components (DAPC) presenting interrelationships based on the genotyping data within twelve microsatellite loci of 143 *V. inaequalis* isolates sampled in two apple growing regions in Latvia and six reference strains with known virulence. Coloured dots enclosed with 95% confidence ellipses represent isolates included in the genetic clusters (K = 4) that were identified in STRUCTURE analysis. The graphs show eigenvalues retained in the discriminant analysis (DA) and principal component analysis (PCA).

Genetic discrimination of the Latvian *V. inaequalis* population into four (K = 4; with reference strains) or three (K = 3; without reference strains) genetic groups, revealed by the population structure analysis, was generally confirmed by PCA and DAPC (Figure 3 and Figure 4).

The components one (PC1, 52.0%) and two (PC2, 33.8%) in the DAPC of populations (K = 4) exposed in the bi-dimensional plot (Figure 4) elucidated the majority of variations between groups. Groups K2 and K3 exhibited a large degree of overlap, thus indicating similarity. The deeper analysis of the strains of each group (K = 3) revealed that in the overlapping fields and in the central part of the DAPC plot, there are strains that were sampled equally from each group and were placed in the largest group, K3, in the second population structure analysis (K = 4) (Figure 3 and Figure 4). Groups K1 and K4, which did not include any of the reference strains with known virulence, were the most distant (Figure 4).

## 4. Discussion

Plant pathogens with mixed reproduction systems (sexual and asexual) pose the greatest risk for host adaptation and breaking down the resistance [41]. Due to the life cycle of *V. inaequalis* (annual sexual reproduction during the host dormancy period and several asexual multiplications during the vegetation season), plants are infected with the newly released ascospores each spring; thus, the pathogen population acquires new genotypes, ensuring the high potential for genetic diversity and adaptation ability. The diversity and population structure of *V. inaequalis* has caught the attention of research groups in Europe and other regions for decades [10,13,15,31]. In contrast, in some regions only recently studies on the genetic diversity of this economically important pathogen have been carried out [9,18,42]. This study is the first report about *V. inaequalis* population diversity in Latvia based on the genotyping within twelve microsatellite loci. We used a highly diverse collection of strains, collected in different years, in two main apple-growing regions from various habitats (commercial orchards, germplasm collections, home gardens, and greeneries), host genotypes, and plant protection systems.

The SSR markers used in this study were highly variable (2 to 29 alleles per locus), and markers Vitc 2D (29 alleles per locus), 1tc1g (27 alleles per locus), and 1tc1a (19 alleles per locus) were the most informative in terms of identifying the genetic diversity of the *V. inaequalis* population. In our study, markers Vitc2/D and 1tc1g revealed the greatest polymorphism that coincided with results from other studies. Similarly, in studies from the United Kingdom [22], South Africa [18], five European countries (France, England, Belgium, Switzerland, Germany, Greece, and The Netherlands) [21], and Belarus [20], the Vitc2/D marker had the greatest polymorphism, as it showed 16 to 37 alleles per locus depending on the population. In contrast, in the studies on *V. inaequalis* population diversity in the United States and the North Caucasian region in Russia, this marker showed one of the least amount of polymorphism (six alleles per locus) [2]. Similarly, the marker 1tc1g showed a great polymorphism (48 alleles) in the 11 populations of *V. inaequalis* in Europe [31] but it was the least polymorphic (two alleles per locus) in the *V. inaequalis* population studied in Turkey [17]. Out of the 31 SSR markers tested by us, 19 were excluded from further use because they were monomorphic, they amplified more than one fragment, or they had no amplification. On the contrary, some of the excluded monomorphic markers in other studies have shown a great polymorphism (2–19 alleles per locus) [2,9,17,18,19,20,22]. Out of the 28 SSR markers that were screened for population genotyping in Iran, five were monomorphic—Vitc1/82, viga3/z, viaacs10, vigt8/146, 1aac4h [9]. Markers Vitc1/82 and Vica9/x turned out to be monomorphic in the genotyping of the South African population [18]. In the genotyping of the *V. inaequalis* population in Belarus, only the Vitc2/16 marker was monomorphic for all of the studied strains [20]. The differences between marker polymorphism among the studies in different regions could be explained by differences in the studied populations (e.g., in Europe, the United States, or the North Caucasian region in Russia).

We used two complementary analysis methods, STRUCTURE and PCA, to study the population structure and relationships between *V. inaequalis* populations in Latvia. Our results from both analyses showed that the populations were highly similar in both regions, and the geographical location made no contribution towards the population differentiation of *V. inaequalis.* No differentiation between these regions could be expected due to the distribution of the propagation material from the two main germplasm collections and nurseries located in these regions (Dobele, Zemgale and Pūre, Kurzeme), a relatively short distance (about 100 km) between both germplasm collections, and a long history of material exchange between them. The germplasm collections in both regions have been Latvia’s main cultivar introducers and planting material distributors for decades. The AMOVA analysis showed only 5% of the variance among the populations confirming the frequent gene flow between the regions. Our findings correspond with previous studies demonstrating that *V. inaequalis* in Europe has high genetic diversity within populations but low diversity among populations, which increases with the expansion of spatial distance [10,13,31]. Similar results were also obtained in later studies, in different countries and continents [2,9,16,19,43]. The results of AMOVA analyses, pointing to higher levels of variation within populations than between populations, and the evidence of admixture in STRUCTURE analysis clearly indicated the low contribution of geographical location to population differentiation and free flow of genes between the populations that could be due to the trade in agriculture, ascospore and conidia dispersal via wind. The transportation of trees from nurseries may move inoculums from one area to another over long distances, which shows the importance of humans in influencing the population dynamics of plant pathogenic fungi [1]. The high variation within populations indicates the role of annual sexual reproduction, the heterothallic mating system of *V. inaequalis*, and the great diversity of grown cultivars. Recombination occurs via sexual reproduction, thus leading to the high variation and diversity in fungi, and changes in the genetic structure of populations [21,24,44]. The favourable weather conditions during the winter and spring in Latvia may positively affect annual sexual reproduction, thus resulting in higher genetic diversity within populations. In addition, the high diversity of cultivars may also have a significant differential selection pressure on *V. inaequalis* in orchards [14,42]. Our results were consistent with previous works [9,18,19,42].

The STRUCTURE analysis identified three genetic lineages among the Latvian isolates (K1-K3) that were also supported by DAPC. In general, the distribution of the isolates in these lineages did not clearly correlate with geographic origin, the cultivar from which the pathogen was isolated, organ (leaves or fruits), time of the collection, and type of the isolation (single conidium or ascospore). Four genetic lineages were identified when six reference strains, with known virulence, were included in the population structure analysis to clarify the possible reasons for the grouping of isolates regardless of their origin. The segregation of the strains in the identified groups indicated the possible relatedness to the virulence. The largest group (K3) included reference strains virulent on the *Rvi1*, *Rvi4,* and *Rvi8* hosts. Over 50% of Latvian *V. inaequalis* isolates, clustering in this group, were from Zemgale, commercial orchards with fungicide applications, and isolated from fruits before 2013. The clustering of the strains was possibly related to the virulence, and this is supported by our previous findings. In Zemgale, the *V. inaequalis* races (1), (4), and (8) are established and capable of overcoming the *Malus* resistances genes *Rvi1*, *Rvi4*, and *Rvi8* [45]. This finding is not surprising since races (1) and (8) occur widely in several European countries, whereas race (4) is only established in some countries in Eastern Europe [11,46], where several of the apple cultivars grown in Latvia originate. The other three reference strains, that are virulent on the *Rvi2*, *Rvi5,* and *Rvi6* hosts, clustered together (K2) with the *V. inaequalis* isolates that were mainly obtained from leaves and fungicide-free growing sites (germplasm collection, greenery, home gardens) in the Dobele region (Zemgale). In both groups, K3 and K2, the number of host genotypes (n = 13 and n = 14, respectively) was higher than in other groups (K1 n = 8 and K4 n = 10). The segregation of the *V. inaequalis* population in the orchard, depending on the virulence, has been demonstrated in Germany, where the studied population was split into two subpopulations based on the strain sampling from host genotypes with or without the *Rvi6* gene [47]. The ability of the pathogen to overcome the *Malus* resistance genes *Rvi2*, *Rvi5,* and *Rvi6* had not yet been observed in closely located orchard with *V. inaequalis* races differential hosts in Dobele; however, the first signs of overcoming the *Rvi6* resistance gene were noticed in organic farms in other regions of Latvia [45,48]. In commercial orchards, especially in organic farms, and in home gardens, locally bred cultivars with resistance genes, predominantly *Rvi6*, have been grown in small quantities since 2011 [49,50]. Moreover, intensive resistant apple breeding, including resistance provided by the *Rvi5* and *Rvi6* genes, has been carried out in Latvia since 1989 [50,51,52]. Although there were no isolates from the *Rvi5* or *Rvi6* hosts among the genotyped isolates from Latvia, the virulent pathogen could infect other genotypes without resistance genes, and such sites without scab management (greenery, home gardens), near commercial orchards or nurseries, may be the source of the diversity. The great diversity of the apple cultivars that have different resistance, or orchards only with resistant cultivars, may still require fungicide applications that can contribute to the reduction of virulence build-up in the pathogen’s population and avoid the risk of the breakdown of resistance in genes in resistant cultivars. Although no or insufficient use of plant protection measures, including leaf litter sanitation, may pose a threat in terms of virulence formation in the *V. inaequalis* population [53] and gradual dispersal to other regions by wind-blown spores or the human transportation of infected planting material.

## Figures and Tables

**Table 1:** Number and origin of *V. inaequalis* strains collected in Latvia which were genotyped using microsatellite markers.

Type of Locality and Host Genotype	Number of Localities (Number of Strains), Sprayed/Non-Sprayed/Total		
	Zemgale	Kurzeme	Total
**Commercial orchards**	**3/0/3 (26/0/26)**	**5/0/5 (44/0/44)**	**8/0/8 (70/0/70)**
‘Lobo’	2/0/2 (19/0/19)	2/0/2 (6/0/6)	4/0/4 (25/0/25)
‘Auksis’	1/0/1 (7/0/7)	1/0//1 (2/0//2)	2/0/1 (9/0/9)
‘Kovalenkovskoje’	-	1/0/1 (7/0/7)	1/0/1 (7/0/7)
‘Belorusskoje Malinovoje’	-	2/0/2 (14/0/14)	2/0/2 (14/0/14)
‘Rubin’	-	1/0/1 (6/0/6)	1/0/1 (6/0/6)
*Malus domestica*, unknown	-	2/0/2 (9/0/9)	2/0/2 (9/0/9)
**Home gardens**	**0/4/4 (0/23/23)**	**0/1/1 (0/3/3)**	**0/5/5 (0/26/26)**
‘Huvitus’	0/1/1 (0/11/11)	-	0/1/1 (0/11/11)
‘Sister of Liberty’	0/1/1 (0/1/1)	-	0/1/1 (0/1/1)
‘Antonovka’	0/1/1 (0/2/2)	-	0/1/1 (0/2/2)
‘Auksis’	0/1/1 (0/1/1)	-	0/1/1 (0/1/1)
‘Vista Bella’	0/1/1 (0/1/1)	-	0/1/1 (0/1/1)
‘Rudens Svītrainais’	0/1/1 (0/5/5)	-	01/1 (0/5/5)
*Malus domestica*, unknown	0/1/1 (0/2/2)	0/1/1 (0/3/3)	0/1/1 (0/3/3)
**Germplasm collections**	**1/1/2 (12/17/29)**	**-**	**1/1/2 (12/17/29)**
‘Rāja’	0/1/1 (0/7/7)	-	0/1/1 (0/7/7)
Nr.16-97-109	0/1/1 (0/5/5)	-	0/1/1 (0/5/5)
‘Belorusskoje Malinovoje’	0/1/1 (0/5/5)	-	0/1/1 (0/5/5)
Nr.29-97-1	1/0/1 (6/0/6)	-	1/0/1 (6/0/6)
‘Stars’	1/0/1 (5/0/5)	-	1/0/1 (5/0/5)
‘Popes ābele’	1/0/1 (1/0/1)	-	1/0/1 (1/0/1)
**Greenery**	**0/1/1 (0/18/18)**	**-**	**0/1/1 (0/18/18)**
Columnar apple, unknown	0/1/1 (0/10/10)	-	0/1/1 (0/10/10)
‘Carnikava’	0/1/1 (0/4/4)	-	0/1/1 (0/4/4)
*Malus toringo*	0/1/1 (0/4/4)	-	0/1/1 (0/4/4)
**Total**	**4/6/10 (38/58/96)**	**5/1/6 (44/3/47)**	**9/7/16 (82/61/143)**

**Table 2:** List of microsatellite primers, sequences, allele size ranges, and annealing temperatures used in PCRs for each primer pair in this study.

Locus	Primer Sequences, 5′–3′	Allele Size Range, bp	Annealing Temperature, °C	Reference
Vicacg8/42	F:(FAM-)TGTCAGCCACGCTAGAAG R:CACCGGACGAATCATGC	196–232	60	[21]
Vitg11/70	F:(HEX-)GAAGAGGTTGGAGTGGTTG R:GAACCGAATCTGTACAGGAC	184–196	60	
Vigtg10/95	F:(ROX-)AGGTGTTGCTGTCTTGGAG R:CGATAGTGTCATTTCCAATCC	134–169	58	
Vica9/152	F:(FAM-)GCACCTGCTCTGTCTATCTC R:AAGGTTCAGGCACTGGAG	167–191	58	
Vitc2/D	F:(TAMRA-)GCTCCTTCTGGGTAAGA R:CTCTACATCTCATCCCATC	184–278	58	
Viga7/116	F:(ROX-)GCCTGGTTGTGGATCTGTC R:ATCCTGCTACATCGACCTTC	159–173	60	
Vigt10/ε	F:(ROX-)GCAGTGCAGGAATAGTAAGG R:GCTGTGATACCAGAGAACGA	171–173	60	
Vict1/130	F:(NED-)GATTGGTGACGCATGTGT R:GCTGGAGATTGCGTAGAC	132–152	58	
EMVi029	F:(TAMRA-)ACGAGTCCCAGGTCTCACAG R:TGTTGACGGTCACGGTGTAT	164–248	58 *	[32]
1tc1g	F:(NED-)TCACTCAACAATACAGTTTCTTAG R:TTTCACGGTAGCGATAGGAG	111–185	58	[13]
1tc1b	F:(HEX-)CGATTGGGGATATGAAGACTT R:TTAGTAATCAAATCGCACCCA	149–210	58	
1tc1a	F:(FAM-)TCGAGATCCTCAAACTTCCTT R:TTTTAACTGTGCGGCCTG	109–187	58	

F—forward primer; R—reverse primer. *—modified in the presented work.

**Table 3:** Summary of the genetic analysis of the *V. inaequalis* population in Latvia, genotyped in twelve microsatellite loci.

Locus	Allele Size Range, bp	N_a_	P_a_	N_e_	I	h	PIC
Vicacg8/42	192–226	13	8	2.75	1.42	0.64	0.627
Vitg11/70	186–210	11	6	5.79	1.96	0.83	0.823
Vigtg10/95	128–157	9	4	2.30	1.14	0.57	0.549
Vica9/152	152–185	9	4	2.65	1.42	0.62	0.602
Vitc2D	188–252	29	10	14.31	2.88	0.93	0.934
Viga7/116	139–174	9	3	3.55	1.53	0.72	0.710
Vigt10/ε	174–176	2	1	1.01	0.04	0.01	0.013
Vict1/130	149–157	5	1	1.68	0.87	0.40	0.405
EMVi029	163–218	15	8	4.20	1.91	0.76	0.746
1tc1g	112–188	27	15	9.27	2.63	0.89	0.884
1tc1b	151–191	10	6	2.44	1.31	0.59	0.604
1tc1a	109–160	19	8	13.62	2.73	0.93	0.925

Na—total number of alleles; Pa—private alleles; Ne—effective number of alleles; I—Shannon’s information index; h—gene diversity (Nei); PIC—polymorphic information content.

**Table 4:** Summary of genetic diversity in the three groups of the *V. inaequalis* population in Latvia, identified by STRUCTURE analysis based on the genotyping data within twelve microsatellite loci.

Group	N	N_a_	P_a_	N_e_	k	I	h
K1	54	7.33	1.67	3.60	14	1.33	0.59
K2	40	6.08	0.92	3.71	11	1.18	0.53
K3	49	9.00	2.75	4.57	14	1.51	0.64

N—number of isolates; N_a_—number of different alleles; P_a_—private alleles; N_e_—effective number of alleles; k—number of genotypes; I—Shannon’s information index; h—gene diversity (Nei).

**Table 5:** AMOVA analysis of the molecular variance of *V. inaequalis* from two regions in Latvia, and different genetic groups (K) identified by STRUCTURE analysis based on the genotyping data within twelve microsatellite loci.

Source of Variation	Degrees of Freedom	Sum of Squares	Variance Components	Percentage Variation	PhiPT	Probability
Among populations (Zemgale and Kurzeme)	2	17.33	0.21	5	0.052	0.001
Within population	141	546.74	3.88	95		
Total	143	564.03	4.10	100		
Among populations (STRUCTURE K = 3) *	3	57.41	0.53	13	0.128	0.001
Within population	140	507.65	3.63	87		
Total	143	565.06	4.16	100		
Among populations (STRUCTURE K = 4) **	4	76.10	0.61	15	0.147	0.001
Within Pops	145	511.69	3.53	85		
Total	149	587.79	4.14	100		

PhiPT—genetic differentiation among populations. * Three genetic groups (K = 3) were identified in the first population structure analysis using STRUCTURE software when only strains from Latvia were included. ** Four genetic groups (K = 4) were identified in the second structure analysis using STRUCTURE software when six reference strains with known virulence were included, in addition to isolates from Latvia.

**Table 6:** Summary of the genetic analysis of three groups (K) of the *V. inaequalis* population in Latvia, identified by STRUCTURE analysis based on the genotyping data within twelve microsatellite loci.

Group (K)	Parameter	Vicacg8/42	Vitg11/70	Vigtg10/95	Vica9/152	Vitc2/D	Viga7/116	Vigt10/ε	Vict1/130	EMVi029	1tc1g	1tc1b	1tc1a
K1	N	54	54	54	54	54	54	54	54	54	54	54	54
	N_a_	5	5	6	7	13	6	1	4	8	13	7	13
	N_e_	1.42	2.62	2.12	3.65	8.84	3.88	1.00	1.42	3.70	3.58	3.04	7.92
	I	0.65	1.18	1.05	1.53	2.33	1.50	0.00	0.63	1.65	1.85	1.35	2.25
	h	0.30	0.62	0.53	0.73	0.89	0.74	0.00	0.30	0.73	0.72	0.67	0.87
K2	N	40	40	40	40	40	40	40	40	40	40	40	40
	N_a_	5	6	4	5	13	3	1	2	8	13	3	10
	N_e_	1.71	3.32	2.43	1.53	8.51	2.89	1.00	1.05	5.63	7.69	1.23	7.55
	I	0.85	1.43	1.01	0.74	2.32	1.08	0.00	0.12	1.84	2.25	0.38	2.14
	h	0,42	0.70	0.59	0.35	0.88	0.65	0.00	0.05	0.82	0.87	0.18	0.87
K3	N	49	49	49	49	49	49	49	49	49	49	49	49
	N_a_	9	9	6	7	19	5	2	5	6	20	6	14
	N_e_	2.65	4.95	2.04	2.72	12.84	2.27	1.04	2.99	2.26	9.49	2.57	9.06
	I	1.46	1.81	0.99	1.38	2.72	0.99	0.10	1.31	1.09	2.60	1.24	2.39
	h	0.62	0.80	0.51	0.63	0.92	0.56	0.04	0.67	0.56	0.89	0.61	0.89

N—number of isolates; N_a_—total number of alleles; P_a_—private alleles; N_e_—effective number of alleles; I—Shannon’s information index; h—gene diversity (Nei).

**Table 7:** Summary of genetic diversity based on the genotyping data within twelve microsatellite loci in four groups (K) of V. inaequalis, identified by STRUCTURE analysis, including 143 strains from Latvia and six reference strains with known virulence from other countries.

Group (K)	N	N_a_	P_a_	N_e_	k	I	h
K1	23	4.67	0.42	2.94	7	1.03	0.50
K2	36	8.00	2.00	4.13	14	1.42	0.62
K3	58	7.67	1.83	3.92	14	1.32	0.58
K4	32	5.42	0.75	3.10	9	1.17	0.56

N—number of isolates; N_a_—number of different alleles; P_a_—private alleles; N_e_—effective number of alleles; k –number of genotypes; I—Shannon’s information index; h—gene diversity (Nei).

**Table 8:** Summary of genetic analysis based on the genotyping data within twelve microsatellite loci in four groups (K) of *V. inaequalis,* identified by STRUCTURE analysis, including 143 strains from Latvia and six reference strains with known virulence from other countries.

Group (K)	Parameter	Vicacg8/42	Vitg11/70	Vigtg10/95	Vica9/152	Vitc2/D	Viga7/116	Vigt10/ε	Vict1/130	EMVi029	1tc1g	1tc1b	1tc1a
K1	N	23	23	23	23	23	23	23	23	23	23	23	23
	N_a_	4	5	3	3	9	4	1	2	8	8	3	6
	N_e_	1.44	3.33	1.71	1.43	7.05	2.33	1.00	1.19	5.45	4.52	1.43	4.45
	I	0.64	1.35	0.74	0.56	2.05	0.98	0.00	0.30	1.84	1.74	0.56	1.62
	h	0.31	0.70	0.42	0.30	0.86	0.57	0.00	0.16	0.82	0.78	0.30	0.78
K2	N	36	36	36	36	36	36	36	36	36	36	36	36
	N_a_	10	9	3	6	16	4	1	4	7	17	6	13
	N_e_	2.75	3.70	1.66	2.27	9.00	2.20	1.00	2.81	2.72	11.17	2.87	7.36
	I	1.55	1.70	0.69	1.20	2.47	0.90	0.00	1.20	1.27	2.61	1.26	2.25
	h	0.64	0.73	0.40	0.56	0.89	0.55	0.00	0.64	0.63	0.91	0.65	0.86
K3	N	58	58	58	58	58	58	58	58	58	58	58	58
	N_a_	6	9	4	6	19	3	2	3	8	15	5	12
	N_e_	2.96	4.38	1.57	2.25	10.32	2.15	1.04	1.41	2.91	8.09	1.72	8.25
	I	1.30	1.72	0.65	1.15	2.64	0.85	0.09	0.52	1.43	2.35	0.83	2.27
	h	0.66	0.77	0.36	0.56	0.90	0.54	0.03	0.29	0.66	0.88	0.42	0.88
K4	N	32	32	32	32	32	32	32	32	32	32	32	32
	N_a_	3	5	5	6	10	5	1	4	5	7	4	10
	N_e_	1.14	2.28	2.81	3.58	7.21	3.35	1.00	1.71	3.68	1.88	2.99	5.63
	I	0.28	1.11	1.21	1.45	2.12	1.39	0.00	0.83	1.41	1.07	1.20	2.00
	h	0.12	0.56	0.64	0.72	0.86	0.70	0.00	0.42	0.73	0.47	0.67	0.82

N—number of isolates; N_a_—total number of alleles; P_a_—private alleles; N_e_—effective number of alleles; I—Shannon’s information index; h—gene diversity (Nei).

## Data Availability

Not applicable.

## Supplementary Materials

The following supporting information can be downloaded at: https://www.mdpi.com/article/10.3390/pathogens11101165/s1, Figure S1: Agarose gel electrophoretic profiles of twelve SSR markers. Lanes 1–8: V. inaequalis strains of various origin; Lane C: Water control; Lane M: DNA ladder.
