# Supplementary material for: Genetic Diversity of Venturia inaequalis in Latvia Revealed by Microsatellite Markers
Source: Pathogens. 2022 Oct 9;11(10):1165. doi: 10.3390/pathogens11101165 (PMC9611756; doi:10.3390/pathogens11101165)
Supplement: Supplementary file 1 [file pathogens-11-01165-s001.zip › pathogens-1914968-supplementary.pdf]

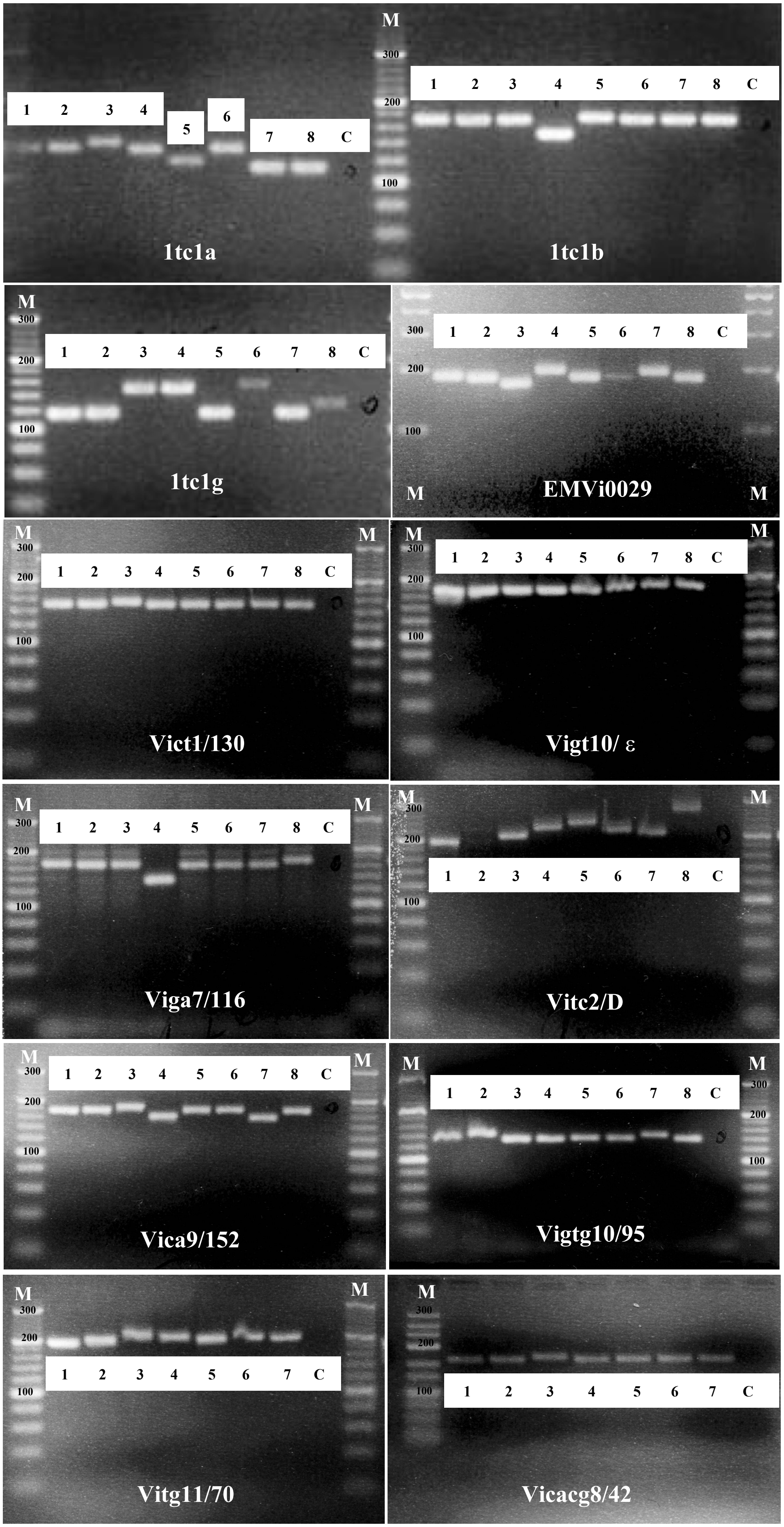

Figure S1. Agarose gel electrophoretic profiles of twelve SSR markers. Lanes 1-8: *V. inaequalis* strains of various origin; Lane C: Water control; Lane M: DNA ladder.
